# System dynamics modelling approach to explore the effect of dog demography on rabies vaccination coverage in Africa

**DOI:** 10.1371/journal.pone.0205884

**Published:** 2018-10-25

**Authors:** Nozyechi Ngulube Chidumayo

**Affiliations:** Clinical Studies Department, School of Veterinary Medicine, University of Zambia, Lusaka, Zambia; Wistar Institute, UNITED STATES

## Abstract

**Background:**

Dogs are important reservoirs of rabies, a zoonotic viral encephalitis that kills thousands of people in Asia and Africa annually. Mass dog vaccination is recommended for the prevention of rabies in both humans and dogs. Mass vaccinations should be conducted annually but more frequent campaigns may be required in areas with high dog turnover rates. Consequently, a good understanding of dog demography is essential for the control of the disease. The aim of this study was to explore the effect of dog demography on rabies vaccination coverage following a mass vaccination campaign with at least 70% vaccination coverage.

**Methodology/Principal findings:**

A dog population model was constructed to explore the effect of dog demography on rabies vaccination coverage decline. Important model parameters were identified through a comprehensive literature search on dog demography in Africa. A system dynamics approach was adopted to build a dog population model to simulate the effects of demographic processes on rabies vaccination coverage. Vensim PLE Plus software was used to construct the model. Multivariate sensitivity simulations using data from 22 studies and 12 African countries were performed to investigate the effect of dog turnover rates on vaccination coverage decline. In addition, an adjusted vaccination coverage to estimate the proportion of vaccinated dogs with adequate immunity at 12 months post-vaccination was calculated. The results demonstrated that the vaccination coverage and adjusted vaccination coverage remained over 30% and 20% respectively at 12 months if annual mass vaccinations achieved at least 70% coverage.

**Conclusions/Significance:**

The results demonstrated that annual mass vaccination campaigns with at least 70% vaccination coverage would maintain a herd immunity of 20‒45% between campaigns.

## Introduction

Rabies is a viral encephalitis transmitted through the saliva of an infected animal [1]. The reservoirs of rabies include domestic dogs, bats and wild carnivores [2–4]. Rabies accounts for up to 55, 000 human deaths annually and the most affected areas are Asia and Africa [5]. The World Health Organisation (WHO) recommends a vaccination coverage of at least 70% to interrupt the transmission cycle [6]. However, the level of vaccination coverage is thought to vary depending on the demographic characteristics of the population [6,7]. Hampson et al. reported that vaccination coverage levels of 20‒45% could interrupt rabies transmission in free-roaming dogs in rural Tanzania [7]. Rabies vaccination provides adequate immunity if dogs develop at least 0.5 IU/ml neutralising antibody titres [6]. Immunity should be maintained for one to three years depending on the vaccine manufacturer and local regulations [8,9]. Antibody titres of vaccinated dogs in Africa vary with some animals failing to seroconvert 30 days after vaccination [10,11]. In addition, some animals develop antibodies 30‒35 days post-vaccination that decline 60‒180 days after vaccination [12,11]. Vaccine efficacy may be compromised as a result of low potency vaccines [13] and immunosuppression due to disease [14,11]

Dog ownership is common in Africa, with 7.7%‒94% dog-owning households [15–23] and an estimated human: dog ratio of 3.7:1‒121:1 [24,22,25,23,20,21,26–28,19,29,30,17,16]. There is generally a male bias [21,29,19,15,31,26,17,20,32,24,30,33,16] that has been attributed to owners preferring male dogs which are considered better at guarding and hunting [25,18]. Dog turnover rates are high and the life expectancy has been reported at 1.7 years [17], 2.9 years [29] and 1.1 years [20]. Most of the dogs in Africa are owned and are therefore available for vaccination [34,35,11,15]. The proportion of ownerless free-roaming dogs has been estimated at <1%‒10.6% [32,17,15,36,37]. The low proportion of ownerless dogs has been attributed to limited food sources to support large populations [17,15]

**Fig 1:**
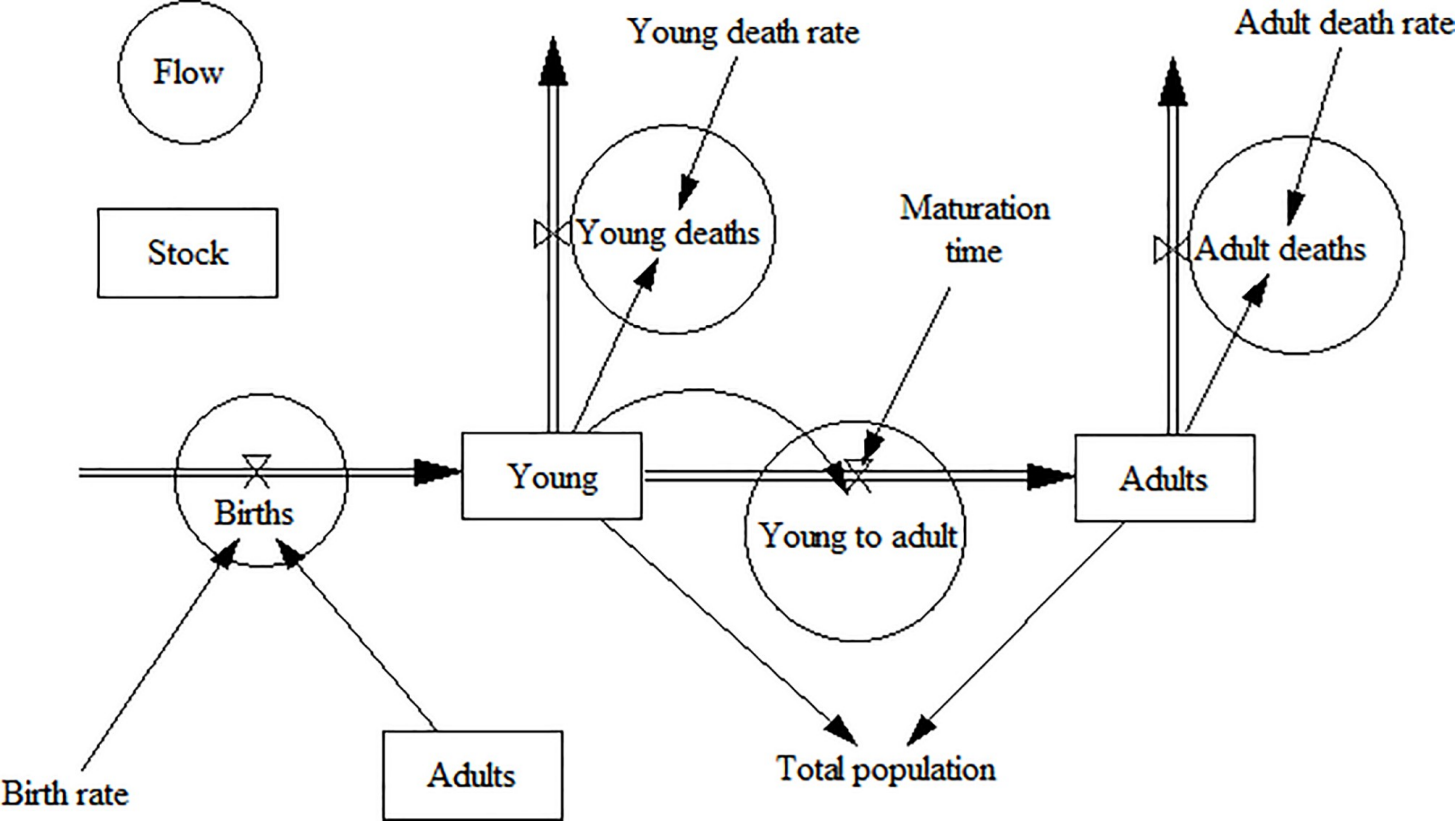
Population stock and flow diagram.

Demography is the study of populations and is concerned with the size, the age and sex composition of the population, and how the population changes over time [38]. Demographic processes, namely births, deaths and migration, affect how populations change over time [38]. Dog demographic processes have an effect on rabies control because they affect vaccination coverage decline. Removal of vaccinated dogs from the population through deaths and emigration can cause a rapid decline in the vaccination coverage [15,18]. This decline may be compounded by the addition of susceptible animals through births and immigration [15,18]. The rate at which the vaccination coverage declines influences how often mass vaccination campaigns should be conducted in an area [15,39]. The WHO recommends annual mass vaccinations with at least 70% vaccination coverage [6]. However, more frequent campaigns may be required for dog populations with high turnover rates [6]. The aim of this study was to assess if annual mass vaccination campaigns with at least 70% vaccination coverage are sufficient to maintain a herd immunity of 20‒45% between campaigns, in dog populations with high turnover rates. Simulation modelling was used to explore the effect of dog demographic processes and vaccine immune response on rabies vaccination coverage decline in dogs in Africa. The influence of demographic processes was assessed using a system dynamics approach. System dynamics is a modelling method that has been used to understand complex environmental [40] and public health [41–44] issues. System dynamics models consist of stock (level) and flow (rate) variables which represent ordinary differential equations [40]. Stocks represent the major accumulations in the system [45]. In a population model, stocks can include the total population or specific subpopulations such as males, females, adults and young. Flows control the rate of change in the stock variables [45]. Inflows and outflows add and deduct from the stocks respectively. In population models, flows can include births, deaths and migrations. Fig 1 shows a population stock and flow diagram with stocks and flows represented by rectangles and circles respectively. The model variables are connected by arrows and mathematical equations are formulated based on the relationships between the variables [45]. For example in Fig 1 the model equations are:

- Births = Adults × Birth rate
- Young to adult = Young ÷ Maturation time
- Young deaths = Young × Young death rate
- Adult deaths = Adults × Adult death rate
- Total population = Young + Adults

## Methods

### Identification of parameters and model development

Literature on dog demography in Africa was reviewed to identify significant model parameters and parameter values. The following data were extracted where possible: the proportion of male dogs; the proportion of female dogs; the proportion of young dogs; the proportion of adult dogs; age-specific mortality rates; mean litter size; the proportion of spayed females and the annual female reproduction probability. For simplicity, dogs aged ≤ 12 months were characterised as young while dogs aged > 12 months were characterised as adults. Age data was further grouped into subpopulations namely: puppies (0–3 months); juveniles (>3 months -12 months); adults 1 (>12 ≤ 24 months); adults 2 (> 24 ≤ 36 months); adults 3 (> 36 ≤ 48 months); adults 4 (> 48 ≤ 60 months); and adult 5 (> 60 months). Table 1 shows the identified model parameters and parameter values.

**Table 1:** Identification of model parameters.

Parameter (Unit)	Value	Citation
Adult proportion (%)	39.4‒79.3	[30,17,29,46,20,26,47,21,15,33]
Female proportion (%)	26.1‒61.2	[16,28,33,30,26,18,32,29,21,20,17,31,23,22,15,19,25,48,24,47,46]
Puppy proportion (%)	5‒34	[15,18,49,17,19]
Juvenile proportion (%)	18, 20, 21.7	[15,17]
Adult 1 proportion (%)	17.7, 14.3,11	[29,17,16]
Adult 2 proportion (%)	15.1, 13.8, 11	[29,17,16]
Adult 3 proportion (%)	7.9, 7.2, 10	[29,17,16]
Adult 4 proportion (%)	2.3, 3.6, 7	[29,17,16]
Adult 5 proportion (%)	6.9, 9.1, 20	[29,17,16]
Puppy death rate (Year^-1^)	0.52‒0.76	[17,19,15,7,31]
Juvenile death rate (Year^-1^)	0.44, 0.65	[15]
Adult 1 death rate (Year^-1^)	0.22, 0.04, 0.0	[29,17,16]
Adult 2 death rate (Year^-1^)	0.33, 0.48, 0.05	[29,17,16]
Adult 3 death rate (Year^-1^)	0.42, 0.50, 0.30	[29,17,16]
Adult 4 death rate (Year^-1^)	0.29, 0.80, 0.77	[29,17,16]
Adult 5 death rate (Year^-1^)	0.29	[29]
Mean litter size	3.8‒6	[31,20,7,32,16,29,19,17,15]
Reproduction probability (Year^-1^)	0.50, 0.55, 0.54	[16,17,29]
Spayed female proportion (%)	0‒5.7	[21,17,29,33,20,16,15]

**Fig 2:**
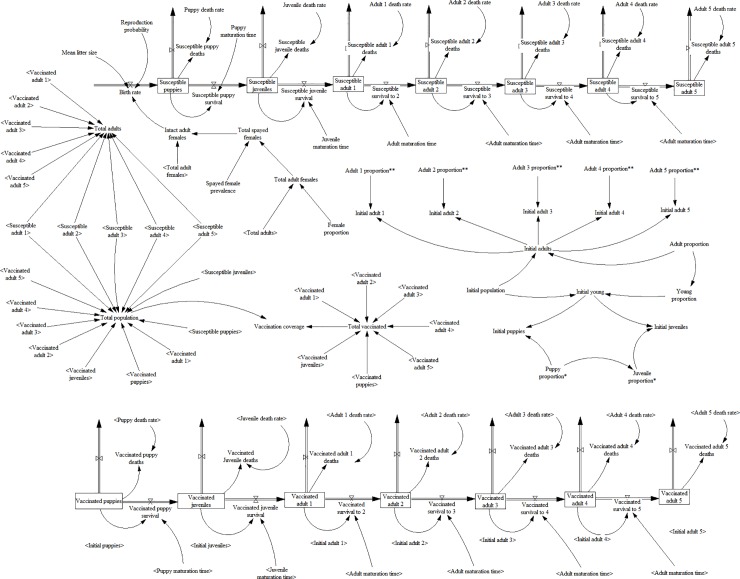
Conceptual model to estimate rabies vaccination coverage.

A stock and flow diagram was constructed based on the parameters identified in Table 1. Vensim PLE Plus (Ventana Systems, 2015) was used to construct the system dynamics model. The model divides the population into susceptible (unvaccinated) and vaccinated dogs. The puppies, juveniles and adults (adults 1‒5) add up to the total population (S1 Dataset: Equation 64) while the puppies and juveniles add up to young dogs. Each compartment has a mortality rate and deaths from each compartment affect the flow of dogs out of the population. The equations for the age-specific deaths are shown in S1 Dataset (Equation 41, 43, 45, 47, 49, 50, 54, 68, 70, 72, 74, 76, 77, 81). Adults are considered to be fertile and therefore contribute to the births that affect the inflow of dogs into the population. Puppies and juveniles are considered to be infertile and therefore do not contribute to the births. The birth rate is the product of the reproduction probability, litter size and the population of reproductive (adult intact) females (S1 Dataset: Equation 14). The model assumes that dogs are born susceptible. The survival rates denote the rate at which dogs in one age group flow into the successive age group (S1 Dataset: Equation 51, 55, 56, 57, 58, 59, 78, 82, 83, 84, 85, 86). The maturation time is the average time for individuals to move from one age class to the next. The puppy, juvenile and adult maturation times are three months, nine months and 12 months respectively. The parts of the model and their relationships are shown in Fig 2. The vaccination coverage is the number of vaccinated dogs divided by the total population (S1 Dataset: Equation 87). The model assumes 100% vaccination of initial juveniles and adults.

### Model validation

Model validation is used to reveal any mathematical and logical flows in the model. Model validation was performed using the boundary adequacy test, structure verification test, parameter verification test, extreme condition test and dimensional consistency test [50–52]. The dimensional consistency of the model was assessed using the Vensim units check function. The model behaviour was evaluated using data from Machakos District, Kenya [29]. This study was selected for model validation because it provided the most information on the model parameter values. The variables used in the model are shown in Table 2. Parameters that affected the births and deaths were selected for model validation (Table 3). To perform the model validation, each selected parameter value was changed while the other parameters remained as shown in Table 2. The model behaviour was then assessed for each scenario and compared to the baseline. The initial population of 5117 dogs was based on the total dog population of Machakos District, Kenya [29].

**Table 2:** Parameter assumptions for model validation based on Kitala et al. [29].

Model parameter	Units	Value
Initial population	Dimensionless	5117
Adult proportion	Dimensionless	0.498
Young proportion	Dimensionless	0.502
Female proportion	Dimensionless	0.403
Puppy proportion *	Dimensionless	0.518
Juvenile proportion *	Dimensionless	0.482
Adult 1 proportion **	Dimensionless	0.355
Adult 2 proportion **	Dimensionless	0.303
Adult 3 proportion **	Dimensionless	0.158
Adult 4 proportion **	Dimensionless	0.046
Adult 5 proportion **	Dimensionless	0.138
Puppy death rate	Month^-1^	0.045
Juvenile death rate	Month^-1^	0.045
Adult 1 death rate	Month^-1^	0.019
Adult 2 death rate	Month^-1^	0.027
Adult 3 death rate	Month^-1^	0.035
Adult 4 death rate	Month^-1^	0.024
Adult 5 death rate	Month^-1^	0.024
Puppy maturation time	Month	3
Juvenile maturation time	Month	9
Adult maturation time	Month	12
Litter size	Dimensionless	4.7
Reproduction probability	Month^-1^	0.045
Spayed female proportion	Dimensionless	0

*Proportion in young dogs

** Proportion in adult dogs

**Table 3:** Parameter assumptions for model validation.

	Scenario		
Parameter	Extreme	Realistic low*	Realistic high**
Adult proportion	0	0.394 [30]	0.793 [33]
Female proportion	0	0.261 [16]	0.612 [46]
Spayed female proportion	1	0 [29]	0.057 [15]
Puppy death rate (Month^-1^)	0	0.044 [17]	0.063 [31]
Juvenile death rate (Month^-1^)	0	0.037 [15]	0.054 [15]
Adult 1 death rate (Month^-1^)	0	0 [16]	0.018 [29]
Adult 2 death rate (Month^-1^)	0	0.004 [16]	0.040 [17]
Adult 3 death rate (Month^-1^)	0	0.025 [16]	0.042 [17]
Adult 4 death rate (Month^-1^)	0	0.024 [29]	0.067 [17]
Adult 5 death rate (Month^-1^)	0		
Litter size	0	3.8 [31]	6 [15]
Reproduction probability	0	0.042 [16]	0.046 [17]

*Parameter values based on the minimum values reported in Table 1

**Parameter values based on the maximum values reported in Table 1

### Sensitivity simulations

Multivariate sensitivity simulations were performed to investigate how sex, age, reproductive probability, neutering, mortality rates and litter size interact and affect vaccination coverage decline. Values for the sensitivity simulations were based on the data extracted from the articles identified in the literature review shown in Table 1. The minimum, median and maximum values were obtained for parameters where data could be extracted from at least five articles. The calculated minimum, median and maximum values were used to generate random numbers for the simulations based on triangular distributions. For parameters where data could be extracted from three or four articles, the minimum and maximum values were used as boundaries for uniform distributions. Parameters where data was reported in less than three articles were excluded from the analysis. Multivariate sensitivity simulations were performed using the Vensim Monte Carlo function. To perform the multivariate sensitivity simulations, parameter values were sampled from within the bounds of the random distributions and the resulting values were used in the simulations. A total of 1000 simulations were performed. The variables used in the simulations are shown in Table 4. The results of the simulations were represented as sensitivity histograms and sensitivity graphs. The sensitivity histogram displayed the number of simulations for which the vaccination coverage was in a given range at 12 months post-vaccination. The sensitivity graph showed the confidence bounds of the vaccination coverage at 12 months post-vaccination.

**Table 4:** Parameter assumptions for sensitivity simulations.

Parameter (citation)	Units	Distribution	Values
Adult proportion [30,17,29,46,20,26,47,21,15,33]	Dimensionless	Random triangular	0.394, 0.645, 0.793*
Female proportion [16,28,33,30,26,18,32,29,21,20,17,31,23,22,15,19,25,48,24,47,46]	Dimensionless	Random triangular	0.261, 0.443, 0.612 *
Puppy death rate [17,19,15,7,31]	Month^-1^	Random triangular	0.044, 0.049, 0.063 *
Adult 1 death rate [29,17,16]	Month^-1^	Random uniform	0.00, 0.018 **
Adult 2 death rate [29,17,16]	Month^-1^	Random uniform	0.004, 0.040 **
Adult 3 death rate [29,17,16]	Month^-1^	Random uniform	0.025, 0.042 **
Adult 4 death rate [29,17,16]	Month^-1^	Random uniform	0.024, 0.067 **
Litter size [31,20,7,32,16,29,19,17,15]	Dimensionless	Random triangular	3.8, 5.4, 6 *
Spayed female proportion [21,17,29,33,20,16,15]	Dimensionless	Random triangular	0, 0.004, 0.057 *
Reproduction probability [16,17,29]	Month^-1^	Random uniform	0.042, 0.046 **

* Minimum, median and maximum values

** Minimum and maximum values

Univariate sensitivity analysis was performed to assess which parameter had the most impact on vaccination coverage decline. To perform univariate simulations, each selected parameter was changed independently, while the other parameters were held as shown in Table 2.

To estimate the proportion of vaccinated dogs with adequate immunity, the vaccination coverage at 12 months was multiplied by the immune proportion. The immune proportion is the proportion of vaccinated dogs with neutralising antibody titres of ≥ 0.5 IU/ml at 12 months post-vaccination. A uniform distribution with minimum and maximum values of 60.6% and 80% was used for the simulations [11,53,54]. The adjusted vaccination coverage is the number of vaccinated dogs that maintained antibody titres at 12 months divided by the total population (S1 Dataset: Equation 1 and S1 Fig).

## Results

### Model validation

**Fig 3:**
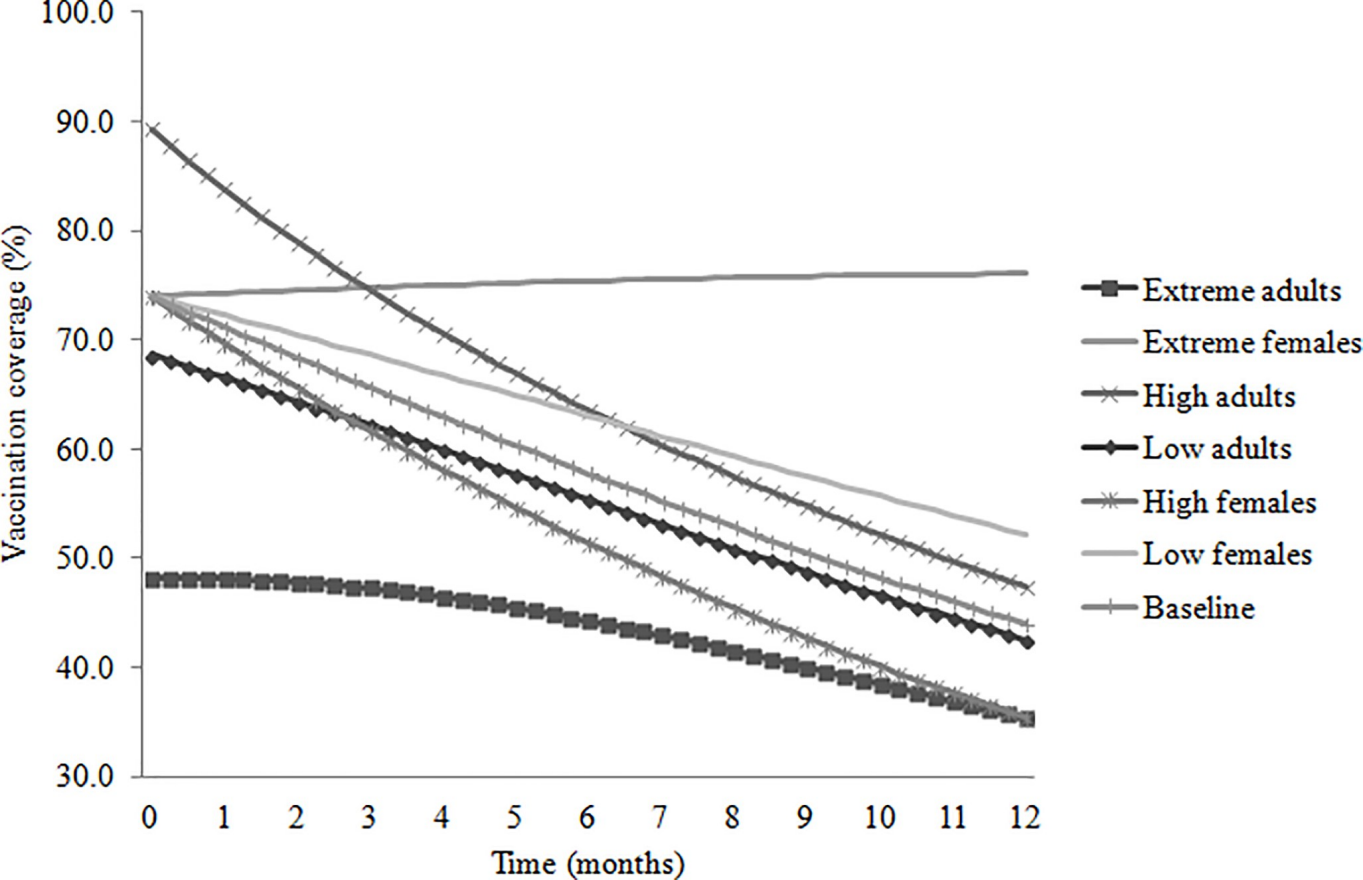
Model validation. Simulation results for five scenarios: baseline; an initial adult proportion of 0% (extreme adults); a female proportion of 0% (extreme females); a female proportion of 26.1% (low females); an initial adult proportion of 39.4% (low adults); a female proportion of 61.2% (high females); and an initial adult proportion of 79.3% (high adults).

Model validation did not reveal any mathematical and logical flows in the model. Fig 3 shows the vaccination coverage output for five scenarios: baseline; an initial adult proportion of 0% (extreme adults); a female proportion of 0% (extreme females); a female proportion of 26.1% (low females); an initial adult proportion of 39.4% (low adults); a female proportion of 61.2% (high females); and an initial adult proportion of 79.3% (high adults). All other variables are as described in Table 2. The vaccination of all juveniles and adults resulted in an initial vaccination coverage of 74% that declined to 44% at 12 months for the baseline. The initial vaccination coverage was lower for the extreme adults and low adults scenarios compared to the baseline while the high adults scenario had a higher initial vaccination coverage compared to the baseline. The vaccination coverage at 12 months was lower for the high females scenario compared to the baseline despite having the same initial vaccination coverage. Conversely, the vaccination coverage at 12 months for the extreme females and low females scenarios was higher than the baseline despite having the same initial vaccination coverage.

### Sensitivity simulations

**Fig 4:**
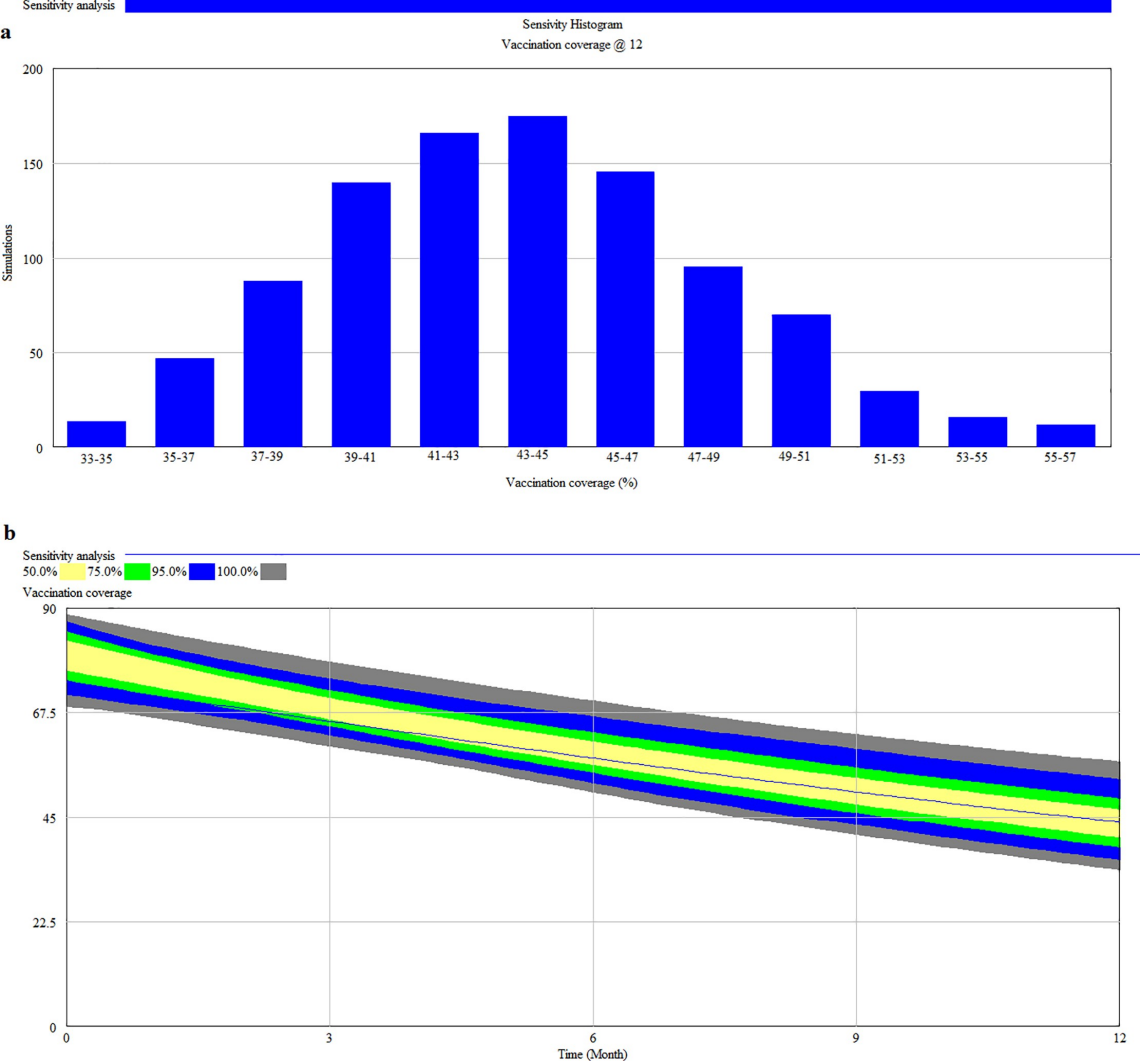
Multivariate sensitivity simulations of rabies vaccination coverage at 12 months post-vaccination. (a) Sensitivity histogram and (b) Sensitivity graph.

**Fig 5:**
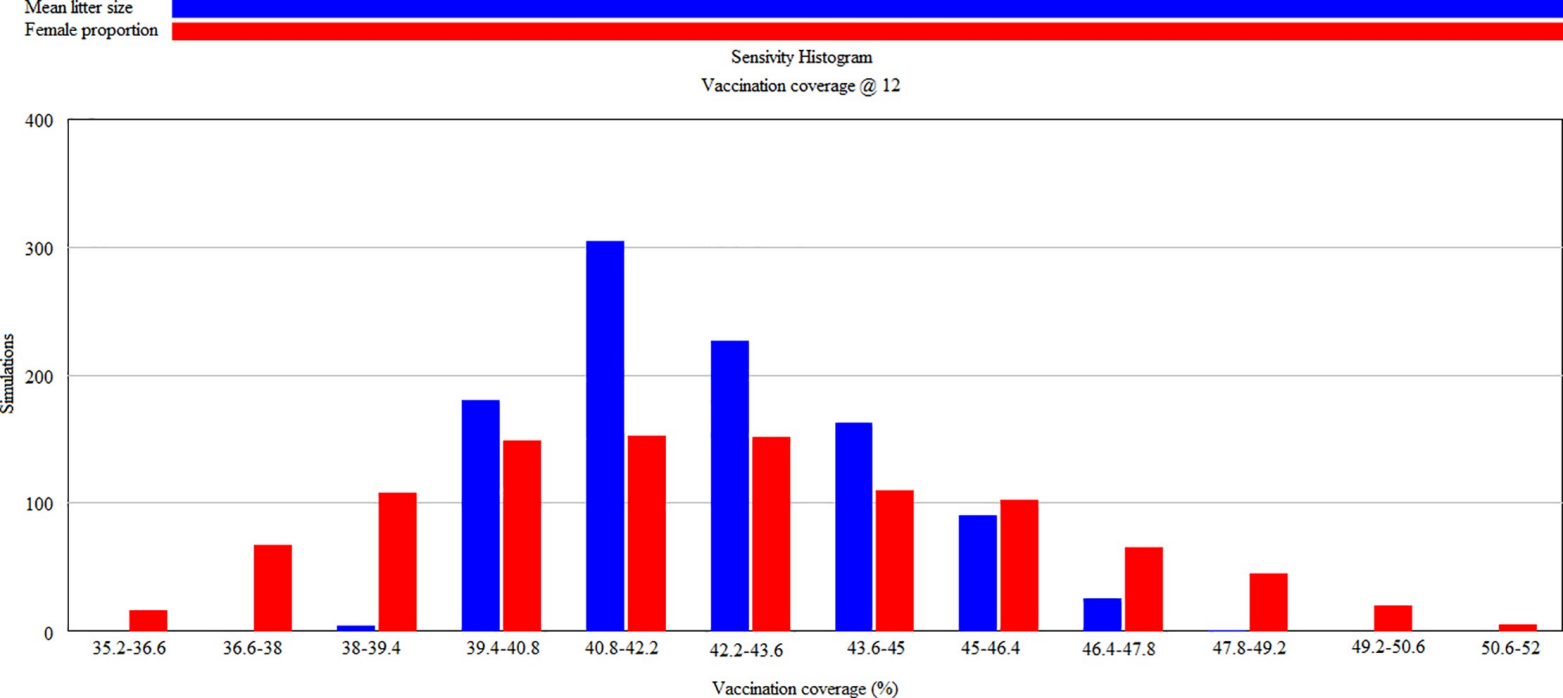
Univariate sensitivity histogram of rabies vaccination coverage at 12 months post-vaccination.

To assess how the interaction of various model parameter values affect vaccination coverage 1000 simulations using ten parameters (Table 4) were performed. Data from 22 studies and 12 countries were included in the analysis. Fig 4A and 4B depict the sensitivity histogram and sensitivity graph results of 1000 simulations at 12 months post-vaccination. Vaccination of all juveniles and adults resulted in an initial vaccination coverage of 68.6‒89.3% and the vaccination coverage remained above 30% at 12 months post-vaccination. Univariate sensitivity simulations showed that the female proportion and mean litter size had the highest impact on vaccination coverage (Fig 5 and S2 Fig).

**Fig 6:**
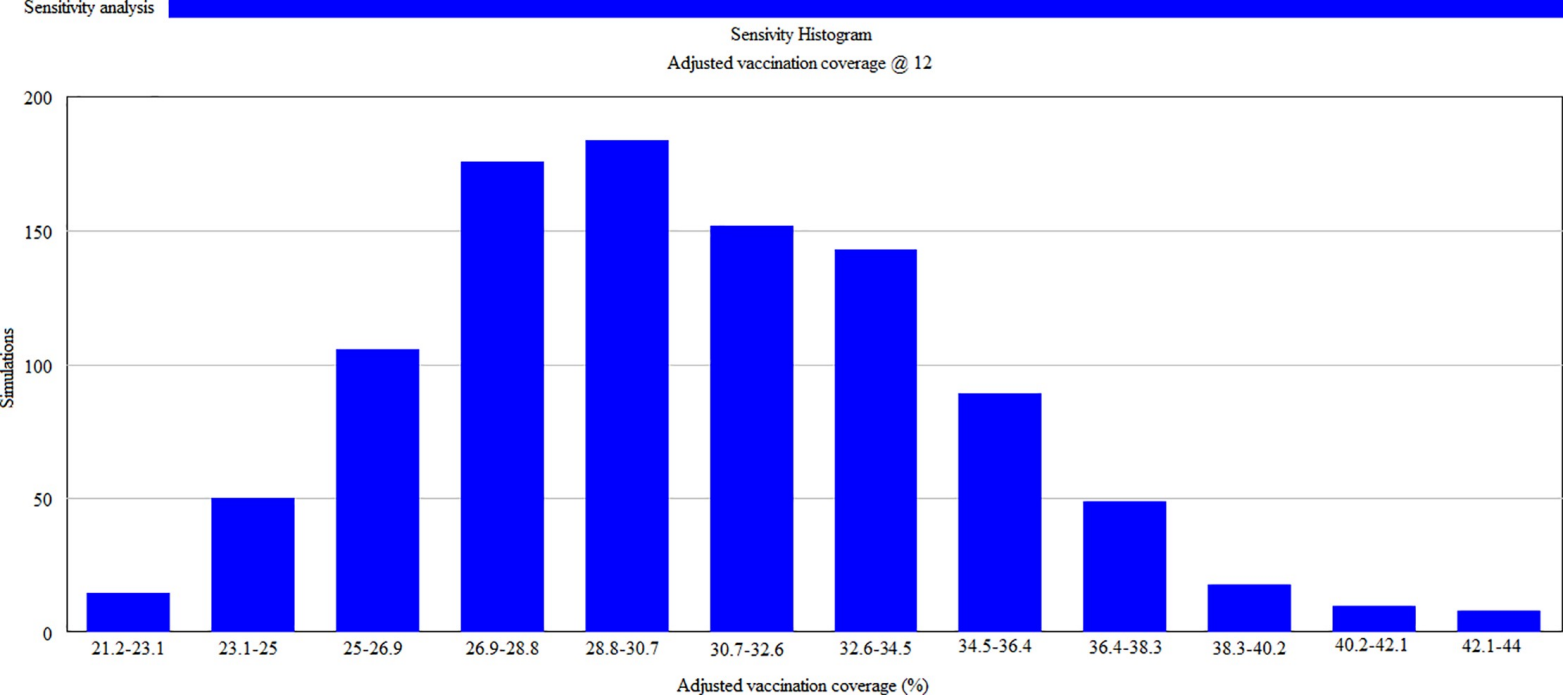
Multivariate sensitivity histogram of the adjusted rabies vaccination coverage at 12 months post-vaccination.

To estimate the proportion of vaccinated dogs with adequate immunity, an adjusted vaccination coverage was calculated. Fig 6 depicts the sensitivity histogram results of 1000 simulations at 12 months post-vaccination. The results showed that the adjusted vaccination coverage remained within 20‒45% at 12 months post-vaccination.

## Discussion

Policy decisions on rabies vaccination campaigns require an understanding of dog demography and how demographic processes interact. This study presents a system dynamics model that explores the effect of dog demographic processes on rabies vaccination coverage decline. A case study of Machakos District demonstrated that an initial mass vaccination coverage of 74% declined to 44% at 12 months post-vaccination. In model validation, the model behaviour for various scenarios was compared with the baseline. Realistically, scenarios with high proportions of unvaccinated puppies due to: high reproduction probabilities; large litter sizes; high proportions of intact adult females; or low puppy mortality are expected to have higher rates of vaccination coverage decline compared to the baseline. Conversely, scenarios with low proportions of unvaccinated puppies due to: low reproduction probabilities; low litter sizes; low proportions of intact adult females; or high puppy mortality would have lower rates of vaccination coverage decline. In the extreme conditions test, the model is expected to generate plausible results outside realistic parameter values. For example, the births should be zero when the female proportion or litter size or reproduction probability is zero as seen in the extreme females scenario. The model behaved in a realistic manner under the various scenarios and the model validation did not reveal mathematical or logical flaws to the model.

Multivariate sensitivity simulations were performed to assess how the interaction of various parameter values would affect the vaccination coverage 12 months after a mass vaccination campaign. The results showed that an initial vaccination of 68.6‒89.3% remained above 30% at 12 months post-vaccination. Moreover, the adjusted vaccination coverage remained within the 20‒45% threshold assuming a seroconversion rate of 60.6‒80%. The results of the model demonstrated that annual mass vaccination campaigns are sufficient to maintain a herd immunity of 20‒45% between vaccination campaigns in dog populations with high turnover rates.

The model assumed that dogs were vaccinated with Rabisin [9] which has been shown to have good seroconversion rates [11,53–56]. The proportion of Rabisin vaccinated dogs with adequate neutralising antibody titres at 12 months post-vaccination has been reported at 60.6%‒80% in Africa [11,53,54], 97% in Peru [56] and 78.4% in Indonesia [11]. Seghaier et al. reported that 36% of dogs vaccinated with a locally produced vaccine had adequate antibody titres 12 months post-vaccination [10]. The model estimated that the adjusted vaccination coverage at 12 months is likely to fall below 20% when the immune proportion is 36% (S3 Fig). It is essential that mass vaccination campaigns use potent vaccines to maintain the desired herd immunity between campaigns. Vaccinations with low potency vaccines result in poor seroconversion rates and/or inadequate persistence of immunity. The use of low potency vaccines would result in a rapid decline in vaccination coverage following a mass vaccination campaign. It is therefore important to conduct studies on the efficacy of locally produced vaccines.

The model did not consider vaccination of puppies since vaccine manufacturers recommend vaccination of dogs at three months of age [9]. However, rabies has been reported in puppies [57–59] and the low vaccination coverage in this age group poses a public health risk given that puppies may represent up to a third of the total population [17,19]. Dogs less than 3 months are usually not vaccinated because they may possess maternal rabies neutralising antibodies that might interfere with the immune response to the vaccine [60]. However, rabies vaccinated puppies in Tanzania and South Africa, demonstrated adequate seroconversion [61]. The WHO recommends that all dogs presented at mass vaccination campaigns should be immunised regardless of age [6]. Vaccination of puppies would, therefore, increase the vaccination coverage in populations with a high proportion of puppies, which is a critical but overlooked variable.

This model has several limitations; firstly, the model does not account for population variations due to the movement of dogs into and out of the population but only considers the population dynamics of the initial population. A high inflow of unvaccinated dogs would increase the rate of vaccination coverage decline. Secondly, the assumption that only dogs over 12 months are fertile is a simplification of the reproductive process. A small proportion of dogs reach fertility at six [32] and ten months [17], however, the vast majority of reproductive dogs are at least 12 months of age [29,17,16]. The exclusion of reproductive dogs under 12 months would underestimate the birth rate and vaccination coverage decline. Another simplification of the model is the assumption that vaccinated and unvaccinated dogs have similar mortality. It has been reported that unvaccinated dogs have a higher mortality rate compared to vaccinated dogs [62]. The assumption that unvaccinated and vaccinated dogs have the same mortality rate may overestimate the rate of vaccination coverage decline. The model only considered owned dogs since the majority of dogs in Africa are owned and therefore available for vaccination [34,35,11,15]. The low proportion of ownerless dogs implies that at least 70% vaccination coverage is attainable by vaccination of owned dogs only. Assuming an ownerless free-roaming dog population of 10%, immunisation of approximately 78% of the owned dog population would achieve at least 70% vaccination coverage. However, baseline data on the owned dog population size is required for adequate human resource mobilisation and procurement of vaccine stocks [63,64]. Unfortunately owned dog populations may be much larger than those reported by officials records [17] and an underestimation of the population could result in a low vaccination coverage. It is important to evaluate the success of vaccination campaigns by conducting post-vaccination studies to estimate the vaccination coverage [64,37,36] and to monitor the occurrence of rabies in dogs and humans [63,65]. Understanding rabies epidemiology in a locality could facilitate targeted vaccination of dogs in areas with the highest rabies transmission rates [63,65]. In addition, targeted vaccination of high risk populations could reduce rabies transmission to adjust areas through incursions of rabid dogs [65].

## Conclusion

This paper employs a system dynamics method to provide an insight on the effect of dog demographic processes on rabies vaccination coverage decline. The results demonstrated that annual vaccination campaigns with at least 70% vaccination coverage would maintain a herd immunity of 20‒45% despite high turnover rates.

## Supporting information

S1 Dataset Model equations. (DOCX)

S1 Fig Conceptual model to estimate adjusted vaccination coverage. (TIF)

S2 Fig Univariate sensitivity histograms of the vaccination coverage at 12 months post-vaccination. (TIF)

S3 Fig Multivariate sensitivity histogram of the adjusted vaccination coverage at 12 months post-vaccination. (TIF)
