# Supplementary material for: System dynamics modelling approach to explore the effect of dog demography on rabies vaccination coverage in Africa
Source: PLoS One. 2018 Oct 25;13(10):e0205884. doi: 10.1371/journal.pone.0205884 (PMC6201891; doi:10.1371/journal.pone.0205884)
Supplement: S1 Dataset — (DOCX) [file pone.0205884.s001.docx]

(01) Adjusted vaccination coverage=

Total immune/Total population

Units: Dimensionless

(02) Adult 1 death rate=

0.019

Units: 1/Month

(03) "Adult 1 proportion**"=

0.355

Units: Dimensionless

** Proportion in adult dogs (the denominator is all adult dogs)

(04) Adult 2 death rate=

0.027

Units: 1/Month

(05) "Adult 2 proportion**"=

0.303

Units: Dimensionless

** Proportion in adult dogs (the denominator is all adult dogs)

(06) Adult 3 death rate=

0.035

Units: 1/Month

(07) "Adult 3 proportion**"=

0.158

Units: Dimensionless

** Proportion in adult dogs (the denominator is all adult dogs)

(08) Adult 4 death rate=

0.024

Units: 1/Month

(09) "Adult 4 proportion**"=

0.046

Units: Dimensionless

** Proportion in adult dogs (the denominator is all adult dogs)

(10) Adult 5 death rate=

0.024

Units: 1/Month

(11) "Adult 5 proportion**"=

0.138

Units: Dimensionless

** Proportion in adult dogs (the denominator is all adult dogs)

(12) Adult maturation time=

12

Units: Month

(13) Adult proportion=

0.498

Units: Dimensionless

(14) Birth rate=

Intact adult females*Mean litter size*Reproduction probability

Units: Dogs/Month

(15) Female proportion=

0.403

Units: Dimensionless

(16) FINAL TIME = 12

Units: Month

The final time for the simulation.

(17) Immune proportion=

0.8

Units: Dimensionless

(18) Initial adult 1=

"Adult 1 proportion**"*Initial adults

Units: Dogs

(19) Initial adult 2=

"Adult 2 proportion**"*Initial adults

Units: Dogs

(20) Initial adult 3=

"Adult 3 proportion**"*Initial adults

Units: Dogs

(21) Initial adult 4=

"Adult 4 proportion**"*Initial adults

Units: Dogs

(22) Initial adult 5=

"Adult 5 proportion**"*Initial adults

Units: Dogs

(23) Initial adults=

Initial population*Adult proportion

Units: Dogs

(24) Initial juveniles=

Initial young*"Juvenile proportion*"

Units: Dogs

(25) Initial population=

5117

Units: Dogs

(26) Initial puppies=

Initial young*"Puppy proportion*"

Units: Dogs

(27) INITIAL TIME = 0

Units: Month

The initial time for the simulation.

(28) Initial young=

Initial population*Young proportion

Units: Dogs

(29) Intact adult females=

Total adult females-Total spayed females

Units: Dogs

(30) Juvenile death rate=

0.045

Units: 1/Month

(31) Juvenile maturation time=

9

Units: Month

(32) "Juvenile proportion*"=

1-"Puppy proportion*"

Units: Dimensionless

*Proportion in young dogs (the denominator is young dogs)

(33) Mean litter size=

4.7

Units: Dimensionless

(34) Puppy death rate=

0.045

Units: 1/Month

(35) Puppy maturation time=

3

Units: Month

(36) "Puppy proportion*"=

0.518

Units: Dimensionless

*Proportion in young dogs (the denominator is young dogs)

(37) Reproduction probability=

0.045

Units: 1/Month

(38) SAVEPER =

TIME STEP

Units: Month [0,?]

The frequency with which output is stored.

(39) Spayed female prevalence=

0

Units: Dimensionless

(40) Susceptible adult 1= INTEG (

Susceptible juvenile survival-Susceptible adult 1 deaths-Susceptible survival to 2

,

0)

Units: Dogs

(41) Susceptible adult 1 deaths=

Susceptible adult 1*Adult 1 death rate

Units: Dogs/Month

(42) Susceptible adult 2= INTEG (

Susceptible survival to 2-Susceptible adult 2 deaths-Susceptible survival to 3

,

0)

Units: Dogs

(43) Susceptible adult 2 deaths=

Adult 2 death rate*Susceptible adult 2

Units: Dogs/Month

(44) Susceptible adult 3= INTEG (

Susceptible survival to 3-Susceptible adult 3 deaths-Susceptible survival to 4

,

0)

Units: Dogs

(45) Susceptible adult 3 deaths=

Susceptible adult 3*Adult 3 death rate

Units: Dogs/Month

(46) Susceptible adult 4= INTEG (

Susceptible survival to 4-Susceptible adult 4 deaths-Susceptible survival to 5

,

0)

Units: Dogs

(47) Susceptible adult 4 deaths=

Susceptible adult 4*Adult 4 death rate

Units: Dogs/Month

(48) Susceptible adult 5= INTEG (

Susceptible survival to 5-Susceptible adult 5 deaths,

0)

Units: Dogs

(49) Susceptible adult 5 deaths=

Adult 5 death rate*Susceptible adult 5

Units: Dogs/Month

(50) Susceptible juvenile deaths=

Susceptible juveniles*Juvenile death rate

Units: Dogs/Month

(51) Susceptible juvenile survival=

Susceptible juveniles/Juvenile maturation time

Units: Dogs/Month

(52) Susceptible juveniles= INTEG (

Susceptible puppy survival-Susceptible juvenile deaths-Susceptible juvenile survival

,

0)

Units: Dogs

(53) Susceptible puppies= INTEG (

Birth rate-Susceptible puppy deaths-Susceptible puppy survival,

Initial puppies)

Units: Dogs

(54) Susceptible puppy deaths=

Susceptible puppies*Puppy death rate

Units: Dogs/Month

(55) Susceptible puppy survival=

Susceptible puppies/Puppy maturation time

Units: Dogs/Month

(56) Susceptible survival to 2=

Susceptible adult 1/Adult maturation time

Units: Dogs/Month

(57) Susceptible survival to 3=

Susceptible adult 2/Adult maturation time

Units: Dogs/Month

(58) Susceptible survival to 4=

Susceptible adult 3/Adult maturation time

Units: Dogs/Month

(59) Susceptible survival to 5=

Susceptible adult 4/Adult maturation time

Units: Dogs/Month

(60) TIME STEP = 0.125

Units: Month [0,?]

The time step for the simulation.

(61) Total adult females=

Female proportion*Total adults

Units: Dogs

(62) Total adults=

Susceptible adult 1+Susceptible adult 2+Susceptible adult 3+Susceptible adult 4

+Susceptible adult 5+Vaccinated adult 1+Vaccinated adult 2+Vaccinated adult 3

+Vaccinated adult 4+Vaccinated adult 5

Units: Dogs

(63) Total immune=

Total vaccinated*Immune proportion

Units: Dogs

(64) Total population=

Susceptible puppies+Susceptible juveniles+Susceptible adult 1+Susceptible adult 2

+Susceptible adult 3+Susceptible adult 4+Susceptible adult 5+Vaccinated puppies

+Vaccinated juveniles+Vaccinated adult 1+Vaccinated adult 2+Vaccinated adult 3

+Vaccinated adult 4+Vaccinated adult 5

Units: Dogs

(65) Total spayed females=

Spayed female prevalence*Total adult females

Units: Dogs

(66) Total vaccinated=

Vaccinated adult 1+Vaccinated adult 2+Vaccinated adult 3+Vaccinated adult 4

+Vaccinated adult 5+Vaccinated juveniles+Vaccinated puppies

Units: Dogs

(67) Vaccinated adult 1= INTEG (

Vaccinated juvenile survival-Vaccinated survival to 2-Vaccinated adult 1 deaths

,

Initial adult 1)

Units: Dogs

(68) Vaccinated adult 1 deaths=

Adult 1 death rate*Vaccinated adult 1

Units: Dogs/Month

(69) Vaccinated adult 2= INTEG (

Vaccinated survival to 2-Vaccinated adult 2 deaths-Vaccinated survival to 3

,

Initial adult 2)

Units: Dogs

(70) Vaccinated adult 2 deaths=

Adult 2 death rate*Vaccinated adult 2

Units: Dogs/Month

(71) Vaccinated adult 3= INTEG (

Vaccinated survival to 3-Vaccinated adult 3 deaths-Vaccinated survival to 4

,

Initial adult 3)

Units: Dogs

(72) Vaccinated adult 3 deaths=

Adult 3 death rate*Vaccinated adult 3

Units: Dogs/Month

(73) Vaccinated adult 4= INTEG (

Vaccinated survival to 4-Vaccinated adult 4 deaths-Vaccinated survival to 5

,

Initial adult 4)

Units: Dogs

(74) Vaccinated adult 4 deaths=

Adult 4 death rate*Vaccinated adult 4

Units: Dogs/Month

(75) Vaccinated adult 5= INTEG (

Vaccinated survival to 5-Vaccinated adult 5 deaths,

Initial adult 5)

Units: Dogs

(76) Vaccinated adult 5 deaths=

Adult 5 death rate*Vaccinated adult 5

Units: Dogs/Month

(77) Vaccinated Juvenile deaths=

Juvenile death rate*Vaccinated juveniles

Units: Dogs/Month

(78) Vaccinated juvenile survival=

Vaccinated juveniles/Juvenile maturation time

Units: Dogs/Month

(79) Vaccinated juveniles= INTEG (

Vaccinated puppy survival-Vaccinated Juvenile deaths-Vaccinated juvenile survival

,

Initial juveniles)

Units: Dogs

(80) Vaccinated puppies= INTEG (

-Vaccinated puppy deaths-Vaccinated puppy survival,

0)

Units: Dogs

(81) Vaccinated puppy deaths=

Vaccinated puppies*Puppy death rate

Units: Dogs/Month

(82) Vaccinated puppy survival=

Vaccinated puppies/Puppy maturation time

Units: Dogs/Month

(83) Vaccinated survival to 2=

Vaccinated adult 1/Adult maturation time

Units: Dogs/Month

(84) Vaccinated survival to 3=

Vaccinated adult 2/Adult maturation time

Units: Dogs/Month

(85) Vaccinated survival to 4=

Vaccinated adult 3/Adult maturation time

Units: Dogs/Month

(86) Vaccinated survival to 5=

Vaccinated adult 4/Adult maturation time

Units: Dogs/Month

(87) Vaccination coverage=

Total vaccinated/Total population

Units: Dimensionless

(88) Young proportion=

1-Adult proportion

Units: Dimensionless
