# Supplementary figures and images for: System dynamics modelling approach to explore the effect of dog demography on rabies vaccination coverage in Africa
Source: PLoS One. 2018 Oct 25;13(10):e0205884. doi: 10.1371/journal.pone.0205884 (PMC6201891; doi:10.1371/journal.pone.0205884)

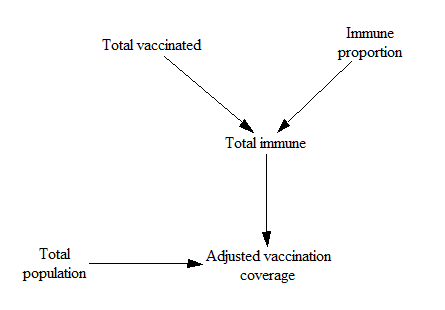

Supplement: S1 Fig — (TIF) [file pone.0205884.s002.tif]

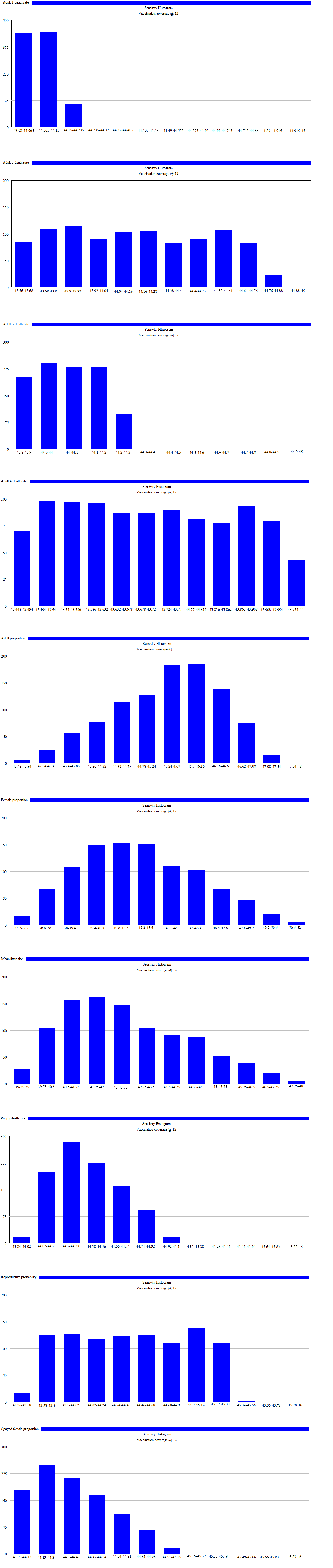

Supplement: S2 Fig — (TIF) [file pone.0205884.s003.tif]

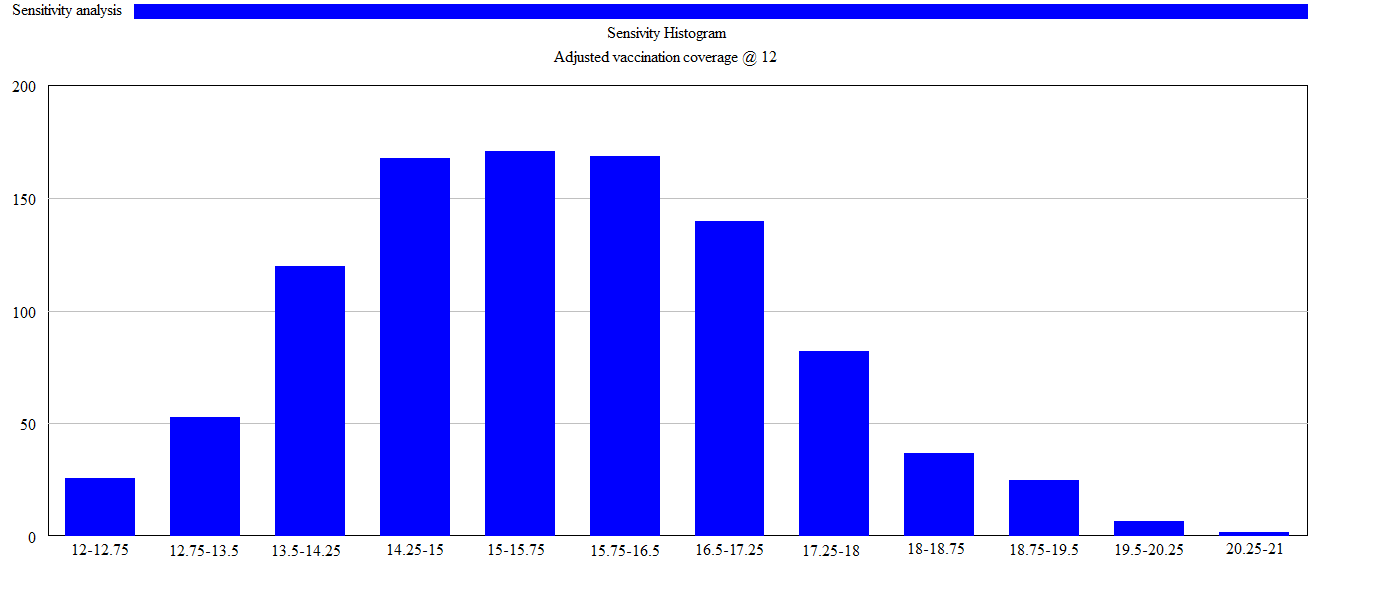

Supplement: S3 Fig — (TIF) [file pone.0205884.s004.tif]
